# Unique Presentation of Urachal Cyst Disease: Incidental Finding to Complicated Infection

**DOI:** 10.1155/2013/874035

**Published:** 2013-04-24

**Authors:** Seong K. Lee, Chauniqua Kiffin, Rafael Sanchez, Eddy Carrillo, Andrew Rosenthal

**Affiliations:** Trauma Services, Memorial Regional Hospital, 3501 Johnson Street, Hollywood, FL 33021, USA

## Abstract

Urachal remnant disease is uncommon in adults and can present with symptoms ranging from drainage near the umbilicus to a severe abdominal infection. Most cases are referred for treatment once diagnosed either clinically or radiographically with ultrasound or computerized tomography. We present a unique case of an infected urachal cyst visualized on a series of CT scans in an adult patient with abdominal pain over a period of years.

## 1. Case Report

**Figure 1:**
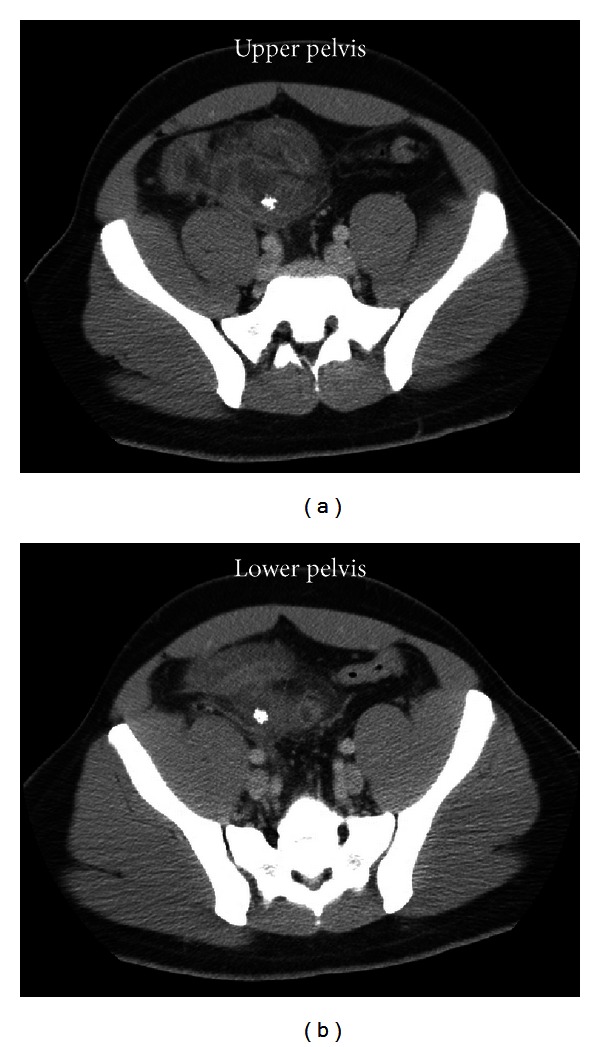
Extensive inflammatory changes in the right lower quadrant associated with an intraperitoneal calcification—common findings in acute appendicitis with a fecalith.

A 33-year-old-male patient presented initially with lower abdominal pain in March 2006. He was diagnosed with appendicitis based upon his exam and history. A CT scan of the abdomen was performed at that time and demonstrated extensive inflammatory changes in the right lower quadrant with possible interloop abscess (Figure 1). Two nonspecific calcifications were noted radiographically and the differential diagnosis included appendicitis, ileitis, or diverticulitis. He did not have any urinary complaints at that time. He underwent an uneventful open appendectomy and had resolution of his initial symptoms and no further follow up. 

**Figure 2:**
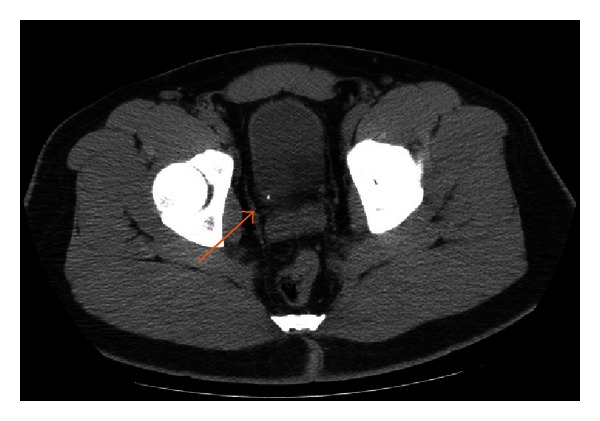
Right uretero-vesicular junction calculus (arrow) associated with right hydroureter (not shown).

**Figure 3:**
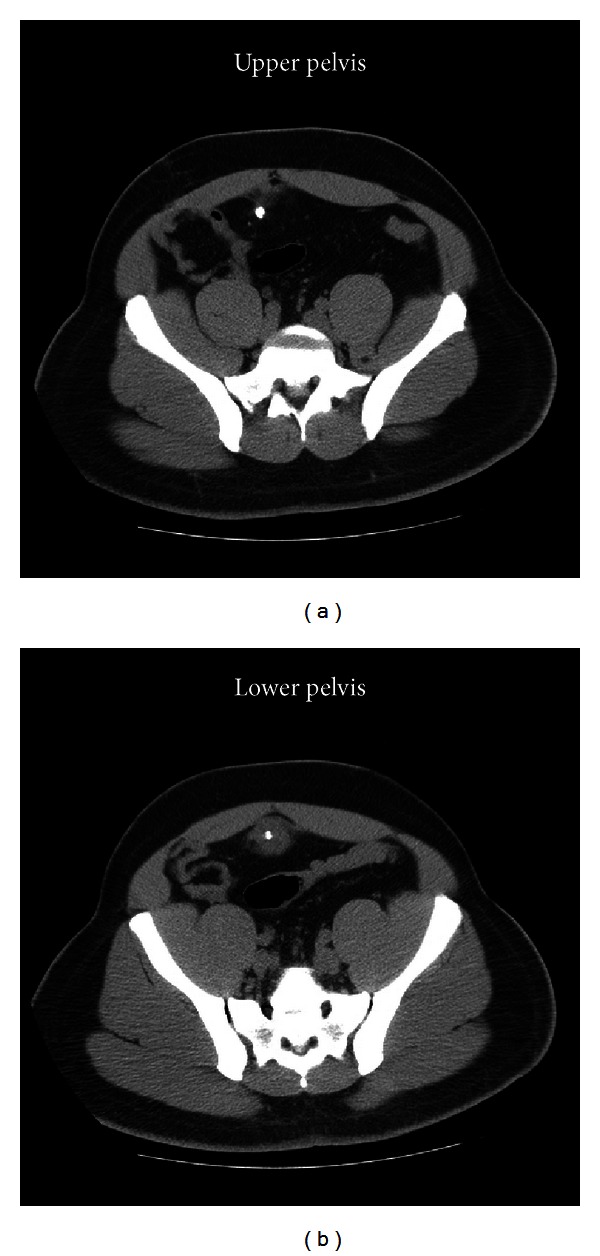
Incidental urachal cyst with calcification without any surrounding inflammation.

The patient presented again in August 2011 with right flank pain and urinary symptoms. A noncontrast CT scan of the abdomen was performed and demonstrated a right ureterovesicular junction calculus with mild right ureteral dilation (Figure 2). Again, calcifications were noted near the anterior fundus of the bladder (Figure 3). He was managed medically and discharged with instructions to follow up with an urologist as an outpatient.

**Figure 4:**
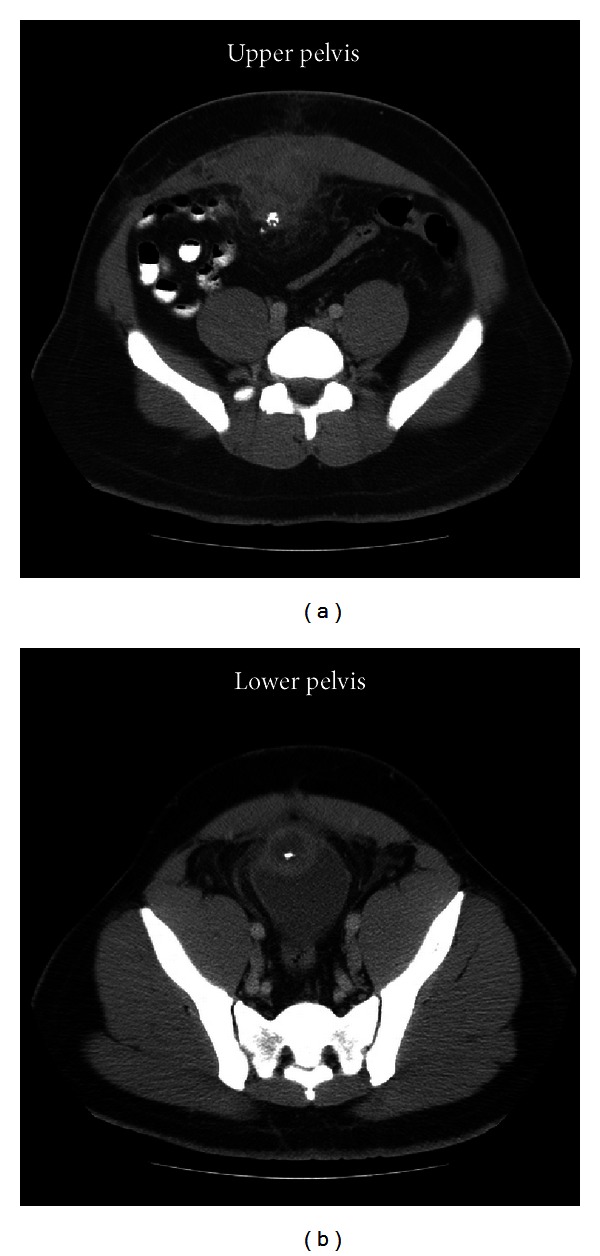
Cystic mass with associated thickening and inflammation adjacent to the abdominal wall and urinary bladder.

He subsequently presented again 3 weeks later with severe abdominal pain and induration of his lower abdominal wall. CT imaging now demonstrated interval enlargement of a complex cystic mass inseparable from the anterior superior wall of the urinary balder and abutting the posterior margin of the abdominal wall (Figure 4). The radiographic diagnosis was an urachal remnant abscess versus tumor. He denied any urologic complaints at that time. He was started on antibiotics, and surgical consultation was performed. After an extensive discussion with the patient regarding his radiographic diagnosis and suspicion of complications related to an urachal remnant, he was scheduled for cystoscopy and surgical exploration. 

At the time of surgery, cystoscopy did not demonstrate any abnormalities of the bladder. Exploratory laparotomy was performed and an indurated mass and abscess were encountered near the area of the anterior abdominal wall and bladder. The abscess was drained and a few small calcifications were encountered and removed for pathologic examination. A complete exploration was performed and the phlegmon appeared to be limited to the dome of the bladder. This area was excised and the dome of the bladder was closed primarily in layers with absorbable suture. Drains were placed and the abdominal wall was debrided and closed. He had an uncomplicated postoperative course. Final pathology demonstrated urachal remnants with no evidence of malignancy. 

## 2. Discussion

The urachus is an embryologic tract that extends from the bladder to the umbilicus during development [1]. Remnants of the tract may present as a patent urachus, vesicourachal diverticulum, urachal sinus, or urachal cyst [1, 2]. A urachal cyst usually presents as an infection in adult patients; however, malignancy has also been reported [2]. An infected urachal cyst usually presents with lower abdominal pain, a tender mass, fever, dysuria, voiding difficulty, or even with umbilical drainage [3]. Diagnosis is suspected when a cystic mass is visualized in the anterior abdominal wall or adjacent to the bladder dome on ultrasound or CT scan. Cysts are usually associated with calcifications. Treatment involves drainage of the infected abscess and excision of the tract including bladder dome if necessary [3].

Our case highlights the potential complications related to an urachal cyst disease in one patient who was treated for multiple causes of lower abdominal pain. The initial presentation of appendicitis was the first glimpse of the cystic remnant disease. The lower abdominal inflammatory changes were nonspecific and therefore it was impossible to differentiate perforated appendicitis with a fecalith from a less common cause of infection. Since the operative and pathologic findings were consistent with appendicitis at that time, consideration of a simultaneous condition was not considered.

Later when the patient presented with a ureterovesicular stone and mild hydronephrosis, the CT scan demonstrated the proximity of the lower abdominal calcifications to the bladder. However, at that time there was no obvious inflammatory changes or infection associated with the bladder or abdominal wall and his symptoms were more consistent with nephroureter obstruction. Despite the radiographic presence of the urachal cyst each time the patient was evaluated for his symptoms, he was diagnosed with a potentially more common cause of pain each time. It was not until the patient developed a severe infection of his lower abdominal wall that the urachal cyst was finally treated surgically and excised. 

## 3. Conclusion

Urachal remnant disease should be considered in any patient with calcifications or cysts adjacent to the anterior abdominal wall or bladder dome. Urachal disease can be an incidental finding radiographically or it may present once infection or some other complication develops. Our case presentation highlights the potential for early diagnosis or potential misdiagnosis due to the frequent use of computerized tomography for patients with lower abdominal pain.
